# Preoperative anaemia and red blood cell transfusion in patients with aneurysmal subarachnoid and intracerebral haemorrhage — a multicentre subanalysis of the German PBM Network Registry

**DOI:** 10.1007/s00701-022-05144-7

**Published:** 2022-02-26

**Authors:** Elke Schmitt, Patrick Meybohm, Vanessa Neef, Peter Baumgarten, Alexandra Bayer, Suma Choorapoikayil, Patrick Friederich, Jens Friedrich, Christof Geisen, Erdem Güresir, Matthias Grünewald, Martin Gutjahr, Philipp Helmer, Eva Herrmann, Markus Müller, Diana Narita, Ansgar Raadts, Klaus Schwendner, Erhard Seifried, Patrick Stark, Andrea U. Steinbicker, Josef Thoma, Markus Velten, Henry Weigt, Christoph Wiesenack, Maria Wittmann, Kai Zacharowski, Florian Piekarski, German PBM Network Collaborators

**Affiliations:** 1 Department of Anaesthesiology, Intensive Care Medicine and Pain Therapy, University Hospital Frankfurt, Goethe University, Frankfurt, Germany; 2 Institute of Biostatistics and Mathematical Modelling, Department of Medicine, Goethe University, Frankfurt, Germany; 3 Department of Anaesthesiology, Intensive Care, Emergency and Pain Medicine, University Hospital Wuerzburg, Wuerzburg, Germany; 4 Department of Neurosurgery, University Hospital Frankfurt, Frankfurt am Main, Germany; 5 Department of Neurosurgery, University Hospital, Schiller University Jena, Jena, Germany; 6 Department of Anesthesiology and Intensive Care Medicine, Krankenhaus Agatharied, Hausham, Academic Teaching Hospital of the Ludwig-Maximilians University of Munich, Munich, Germany; 7 Department of Anesthesiology, Operative Intensive Care Medicine and Pain Therapy, München Klinik Bogenhausen, Munich, Germany; 8 Department of Anesthesiology and Operative Intensive Care Medicine, Klinikum Leverkusen, Leverkusen, Germany; 9 German Red Cross, Institute for Transfusion Medicine and Immunohematology, German Red Cross Baden-Wuertemberg - Hessen, Goethe University Frankfurt, Frankfurt am Main, Germany; 10 Department of Neurosurgery, University Hospital Bonn, Bonn, Germany; 11 Department of Anaesthesiology and Intensive Care Medicine, University Hospital Schleswig-Holstein, Kiel, Germany; 12 Department of Anesthesiology, Marienhaus Klinikum St. Wendel-Ottweiler, Ottweiler, Germany; 13 Institute for Laboratory Diagnostics and Transfusion Medicine, DonauIsar Klinikum, Deggendorf/Dingolfing/Landau, Germany; 14 Department of Anesthesiology and Intensive Care Medicine, University Hospital Jena, Jena, Germany; 15 Department of Anesthesiology and Operative Intensive Care Medicine, Diakonie hospital Martha-Maria, Nuremberg, Germany; 16 Department of Vascular Surgery, Katholisches Klinikum Koblenz, Koblenz, Germany; 17 Department of Anaesthesiology, Intensive Care and Pain Medicine, University Hospital Muenster, Muenster, Germany; 18 Department of Anesthesiology and Operative Intensive Care Medicine, Ortenau Klinikum Offenburg-Kehl, Offenburg, Germany; 19 Department of Anaesthesiology and Operative Intensive Care Medicine, University Hospital Bonn, Bonn, Germany; 20 Department of Anesthesiology, SLK-Kliniken, Heilbronn, Germany; 21 Department of Anesthesiology, Protestant Deaconess Hospital Freiburg, Freiburg, Germany

**Keywords:** Aneurysmal subarachnoid haemorrhage, Intracerebral haemorrhage, Anaemia, Red blood cell transfusion, Patient blood management

## Abstract

**Purpose:**

Anaemia is common in patients presenting with aneurysmal subarachnoid (aSAH) and intracerebral haemorrhage (ICH). In surgical patients, anaemia was identified as an idenpendent risk factor for postoperative mortality, prolonged hospital length of stay (LOS) and increased risk of red blood cell (RBC) transfusion. This multicentre cohort observation study describes the incidence and effects of preoperative anaemia in this critical patient collective for a 10-year period.

**Methods:**

This multicentre observational study included adult in-hospital surgical patients diagnosed with aSAH or ICH of 21 German hospitals (discharged from 1 January 2010 to 30 September 2020). Descriptive, univariate and multivariate analyses were performed to investigate the incidence and association of preoperative anaemia with RBC transfusion, in-hospital mortality and postoperative complications in patients with aSAH and ICH.

**Results:**

A total of *n* = 9081 patients were analysed (aSAH *n* = 5008; ICH *n* = 4073). Preoperative anaemia was present at 28.3% in aSAH and 40.9% in ICH. RBC transfusion rates were 29.9% in aSAH and 29.3% in ICH. Multivariate analysis revealed that preoperative anaemia is associated with a higher risk for RBC transfusion (*OR* = 3.25 in aSAH, *OR* = 4.16 in ICH, *p* < 0.001), for in-hospital mortality (*OR* = 1.48 in aSAH, *OR* = 1.53 in ICH, *p* < 0.001) and for several postoperative complications.

**Conclusions:**

Preoperative anaemia is associated with increased RBC transfusion rates, in-hospital mortality and postoperative complications in patients with aSAH and ICH.

**Trial registration:**

ClinicalTrials.gov, NCT02147795, https://clinicaltrials.gov/ct2/show/NCT02147795

**Supplementary Information:**

The online version contains supplementary material available at 10.1007/s00701-022-05144-7.

## **Background**

Anaemia is an independent risk factor for postoperative complications, mortality, prolonged hospital length of stay (LOS) and increased risk of red blood cell (RBC) transfusion [24]. The prevalence of anaemia is reported to be 22.8% globally and 26.5-31.5% in patients undergoing surgery [2, 10]. Preoperative anaemia was reported for 5.5% of patients suffering from aneurysmal subarachnoid haemorrhage (aSAH) [8] and for 24.1–25.8% of patients suffering from intracerebral haemorrhage (ICH) [16, 17]. For patients with ICH, anaemia has also been shown to be an independent predictor for unfavourable long-term outcomes a decade ago [17]. Kumar et al. demonstrated that anaemia is common in acute ICH patients and that its presence on admission is an independent predictor of increased ICH volume; contrary to pathophysiological considerations, however, in 2009, they could not demonstrate an effect on increased mortality [16].

The treatment of anaemia in emergency situations usually involves the administration of allogeneic blood products. The administration of RBC transfusions is known to be associated with multiple risks, such as transfusion-related lung injury, haemolytic reactions and transmission of infectious diseases [12]. RBC transfusions in patients undergoing cranial surgery are also associated with a prolonged LOS, more postoperative complications, a 30-day return to the operating theatre and an increased 30-day mortality rate [4]. In patients with aSAH, RBC transfusions have been shown to result in increased mortality and general worse clinical outcomes [31].

This multicentre cohort study analyses the incidence of preoperative anaemia and its association with RBC transfusion requirements, hospital length of stay (LOS), in-hospital mortality and clinically relevant outcomes in patients with aSAH and ICH.

## Methods

### Study design and objectives

The current study is a subanalysis of the ongoing prospective multicentre observational study ‘Safety and effectiveness of a Patient Blood Management (PBM) programme in surgical patients’ (ClinicalTrials.gov, NCT02147795) [22]. The period analysed covered 1 January 2010 to 30 September 2020. Data from 23 hospitals was screened. The study was approved by the Ethics Committee of the University Hospital Frankfurt, Goethe University (first vote ref. 380/12 from 10 January 2013, amendments from 17 June 2013 to 1 June 2016, second vote ref. 318/17 from 30 November 2017) who waived the requirement for informed patient’s consent. In addition, the local ethics committee of each participating centre followed this vote and likewise waived the requirement for informed patient’s consent.

The primary objective of the study was to assess the prevalence of preoperative anaemia and its association with RBC transfusion in aSAH or ICH patients. The secondary objective was to investigate the association of potential risk factors (such as preoperative anaemia, RBC transfusions and other factors related with the type of neurosurgical intervention, additional diagnoses and patient characteristics) with common clinical outcomes (including mortality, typical postoperative complications and LOS) in patients with aSAH and ICH (Online Resource 1).

### Patient enrolment and inclusion criteria

**Fig. 1:**
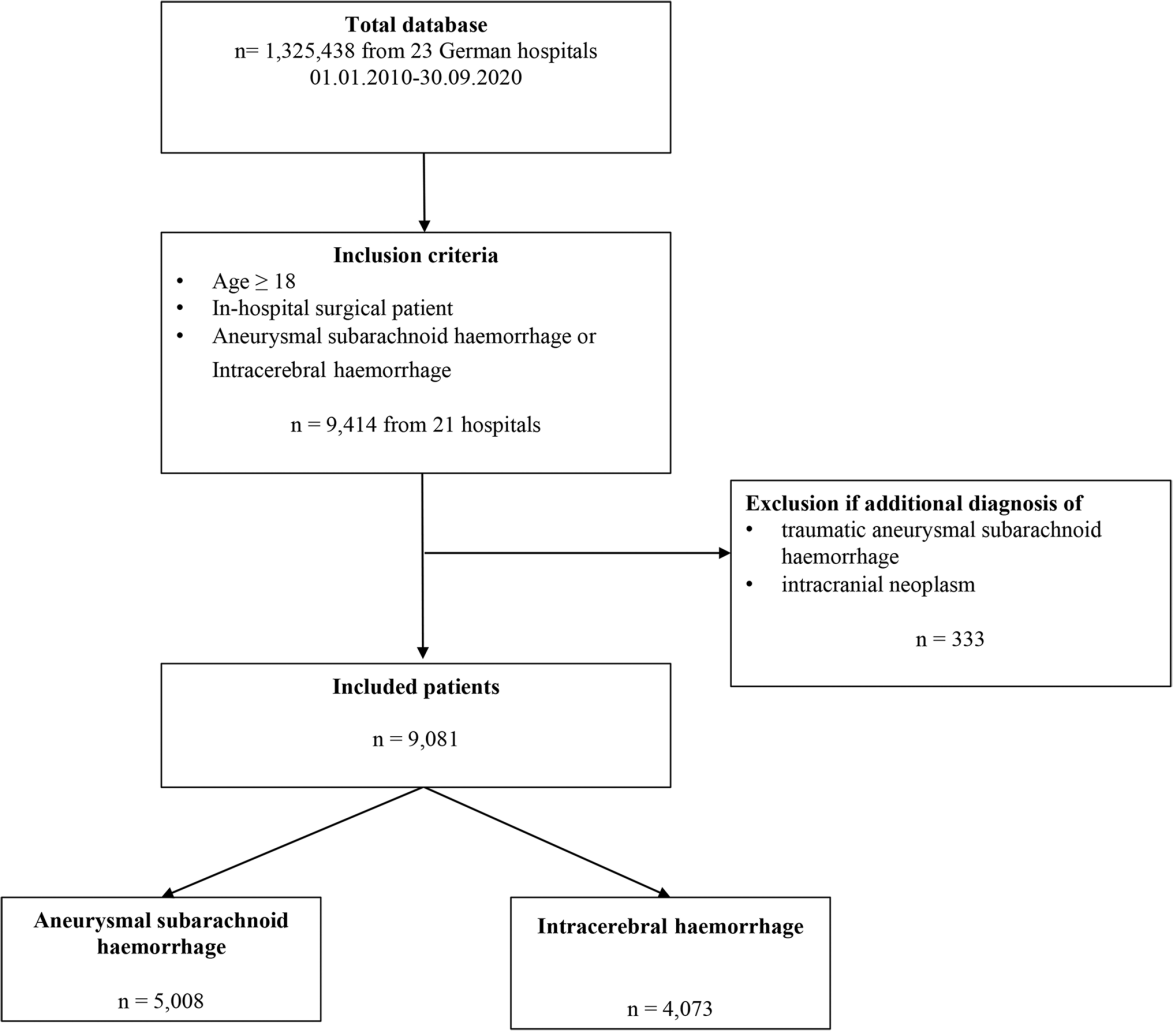
The inclusion and exclusion criteria among patients analysed

The underlying PBM database was contained by design adult (≥ 18 years) in-hospital patients, who underwent surgery or a procedure (classified according to the Operation and Procedure Classification System (OPS) code (Online Resource 1)) during their hospital stay. Patients from the PBM database with a diagnosis of aSAH or ICH, defined by the International Classification of Disease (ICD-10) codes and discharged from hospital within the time period from 1 January 2010 to 30 September 2020, were included (Online Resource 1 and 2). The exclusion criteria were the diagnoses of additional traumatic SAH and intracranial neoplasm (Online Resource 1). Patients were assigned to either the aSAH (patients diagnosed with aSAH with/without additional ICH) or the ICH group (patients diagnosed with ICH only) (Fig. 1). This classification was chosen because an additional ICH can occur after the aetiological event of an aSAH, even though the patients were originally diagnosed only with aSAH. In the group of patients diagnosed with ICH only, ICH was the primary diagnosis and cause of hospitalisation.

### Definitions

Anaemia was defined according to the WHO definition: anaemia Hb < 12 g/dl (7.45 mmol/l) (female) and Hb < 13 g/dl (8.07 mmol/l) (male); mild anaemia in female: Hb 11-12 g/dl (6.83-7.45 mmol/l); mild anaemia in male: Hb 11-13 g/dl (6.83-8.07 mmol/l); moderate anaemia in female and male: Hb 8-11 g/dl (4.97-6.83 mmol/l); severe anaemia in female and male Hb < 8 g/dl (< 4.97 mmol/l) [27].

The preoperative anaemia status was based on the first available preoperative Hb value, and the postoperative anaemia status was based on the last available Hb value before hospital discharge. Diagnostic criteria were defined by the relevant ICD-10 codes (Online Resource S1). Vasospasm was defined by ICD code I67.80. Interventions were defined by the relevant Operation and Procedure Classification (OPS) codes (Online Resource S1). Mortality was defined by the discharge code. Hospital LOS was defined by the given admission and discharge dates.

### Data collection

The underlying data source was an anonymous routine data from hospital information systems (e.g. Agfa Orbis, Nexus, iMedOne, SAP) and additional data from individual blood bank and pharmacy software systems of the corresponding hospitals participating in the epidemiological research and quality management study of the German Patient Blood Management Network [22]. The data transferred to the PBM Network Coordination Centre did not contain any personal information. A data protection vote from the Hessian data protection officer was obtained (ref: 43.60; 60.01.21-ga from 24 October 2018). The biostatistician in charge subsequently evaluated the data for completeness and correctness and extracted the cases that fulfilled the inclusion criteria for this study before performing the final analysis.

### Statistical analysis

Descriptive analysis was used to determine patient characteristics, the prevalence of pre- and postoperative anaemia, RBC transfusions, surgical interventions and postoperative outcomes. The results of the descriptive analysis are presented as means (± standard errors), medians (with first and third quartiles) and rates (with 95% *CI*).

Multivariate mixed effect regression analysis was performed to identify independent predictors of RBC transfusion and various postoperative outcomes. The multivariate mixed effect regression models included the hospitals as random effects (to account for hospital individual effects) and other potentially relevant factors (such as age, gender, surgical interventions, preoperative anaemia, RBC consumption and vasospasm) as fixed effects.

Univariate non-parametric analysis (chi-square tests for binary endpoints and Wilcoxon-Mann-Whitney tests for continuous endpoints) was performed a priori to assess the correlation of the individual factors where appropriate. To account for the heterogeneity of the aSAH and ICH groups, all analyses (univariate and multivariate) were performed separately by group. All analyses were performed using the free software R (version 3.6.3).

## Results

A total of *n* = 1,325,438 patients from 23 hospitals were screened. Two hospitals in the network did not treat patients with neurosurgical diagnoses. Overall, *n* = 9081 eligible patients from 21 hospitals were included and analysed in this study. The aSAH group included *n* = 5008 patients and the ICH group included *n* = 4073 patients (Fig. 1). The incidence of eligible cases within the entire database of *n* = 1,325,438 was 0.4% for aSAH and 0.3% for ICH. Most patients received a neurosurgical OPS (84.9% in aSAH and 76.9% in ICH). The remaining OPS are distributed across several specialties (visceral and endocrine surgery accounts for the highest proportion with 5.0%, followed by 3.5% with otorhinolaryngology). The distribution of other surgical OPS can be found in Online Resource 2). Demographic and intervention data are shown in Table 1.

**Table 1:** Patient characteristics, interventions and anaemia prevalence

		Aneurysmal subarachnoid haemorrhage (*n* = 5,008)	Intracerebral haemorrhage (*n* = 4,073)
Age at admission (years)		57.9 ± 0.2, 57.0 (49.0; 68.0), min = 18, max= 96	65.5 ± 0.2, 68.0 (57.0; 77.0), min = 18, max = 97
Gender		Female: 61.7%; *n* = 3088/5008 Male: 38.3%; *n* =1920/5008	Female: 42.4%; *n* = 1727/4073 Male: 57.6%; *n* = 2346/4073
Clipping		36.1% (34.8-37.5%); *n* = 1809/5008	-
Coiling		27.8% (26.5- 29.0%); *n* = 1390/5008	-
Craniotomy		46.6% (45.2-48.0%); *n* = 2335/5008	44.6% (43.1-46.2 %); n = 1817/4,73
Additionally present intracerebral haemorrhage		23.9% (22.8-25.1%), *n* = 1199/5008	-
Vasospasm In preoperative anaemic patients In preoperative non-anaemic patients		13.4% (12.5-14.4%); *n* = 672/5008 9.3% (7.7-11.0%); n= 113/1218 12.4% (11.3-13.6 %); *n* = 383/3086	- - -
Preoperative Hb (g/dl)		13.1 (± 0.0), 13.2 (12.1; 14.4)	12.8 (± 0.0), 13.0 (11.4; 14.3)
Preoperative anaemia In male In female		28.3% (27.0-29.7 %); *n* = 1218/4304 29.5% (27.3-31.8 %); *n* = 488/1652 27.5% (25.8-29.3 %); *n* = 730/2652	40.9% (39.3-42.6 %); *n* = 1421/3474 42.5% (40.4-44.7 %); *n* = 855//2010 38.7% (36.2-41.2 %); *n* = 566/1464
Preoperative anaemia levels	None	71.7%; *n* = 3086/4304	59.1%; *n* = 2053/3474
	Mild	16.6%; *n* = 713/4304	20.6%; *n* = 716/3474
	Moderate	10.7%; *n* = 462/4304	17.6%; *n* = 610/3474
	Severe	1.0%; *n* = 43/4304	2.7%; *n* = 95/3474
Postoperative Hb (g/dl)		10.7 (± 0.0), 10.5 (9.3; 11.9)	10.5 (± 0.0), 10.3 (8.9; 11.9)
Postoperative anaemia In preoperative anaemic patients In preoperative non-anaemic patients		80.9% (79.8-82.0%); *n* = 3898/4818 95.3% (94.0-96.4%); *n* = 1142/1218 77.1% (75.5-78.6%); *n* = 2334/3086	83.1% (81.9-84.3%); *n* = 3275/3659 96.0% (94.9-97.0%); *n* = 1349/1421 73.1% (71.1-75.1%); *n* = 1478/2053
Hospital-acquired anaemia		77.1% (75.5-78.6%); *n* = 2334/3028	73.1% (71.1-75.1%); *n* = 1478/2021
Haemorrhagic diathesis due to coumarins		1.3% (1.0-1.6%); *n* = 63/5008	4.2% (3.6-4.9%); *n* = 173/4073
Haemorrhagic diathesis due to heparins		0.7% (0.5-0.9%); *n* = 34/5008	0.7% (0.5-1.0%); *n* = 28/4073
Haemorrhagic diathesis due to NOACs		1.2% (0.9-1.6%); *n* = 61/5008	4.4% (3.8-5.1%); *n* = 180/4073
Factor XIII deficiency		0.3% (0.1-0.4%); *n* = 13/5008	0.6% (0.4-0.9%); *n* = 25/4073
Factor VIII deficiency		0.5% (0.3-0.7%); *n* = 23/5008	0.4% (0.2-0.6%); *n* = 15/4073
Left Ventricular Assist Device		0.2% (0.1-0.3%); *n* = 8/5008	0.5% (0.3-0.8%); *n* = 21/4073
Extracorporeal membrane oxygenation		0.7% (0.5-0.9%); *n* = 33/5008	0.7% (0.4-1.0%); *n* = 27/4073
Extracorporeal life support		0.3% (0.2-0.5%); *n* = 16/5008	0.6% (0.4-0.9%); *n* = 25/4073

Table 1 shows the patient characteristics, interventions and anaemia prevalence. Anaemia rates are calculated only from the subset of patients, of whom the required pre- and/or postoperative Hb values were available. The exact numbers are given per individual line. All values are represented as mean (± SE), median (IQR) or as rate (95% CI) and total number

### Anaemia

The median preoperative Hb level was 13.2 g/dl in aSAH patients and 12.8 g/dl in ICH patients. Severe, moderate and mild preoperative anaemia was present in aSAH patients at rates of 1.0%, 10.7% and 16.6%, respectively and in ICH patients at rates of 2.7%, 17.6% and 20.6%, respectively (Table 1).

**Fig. 2:**
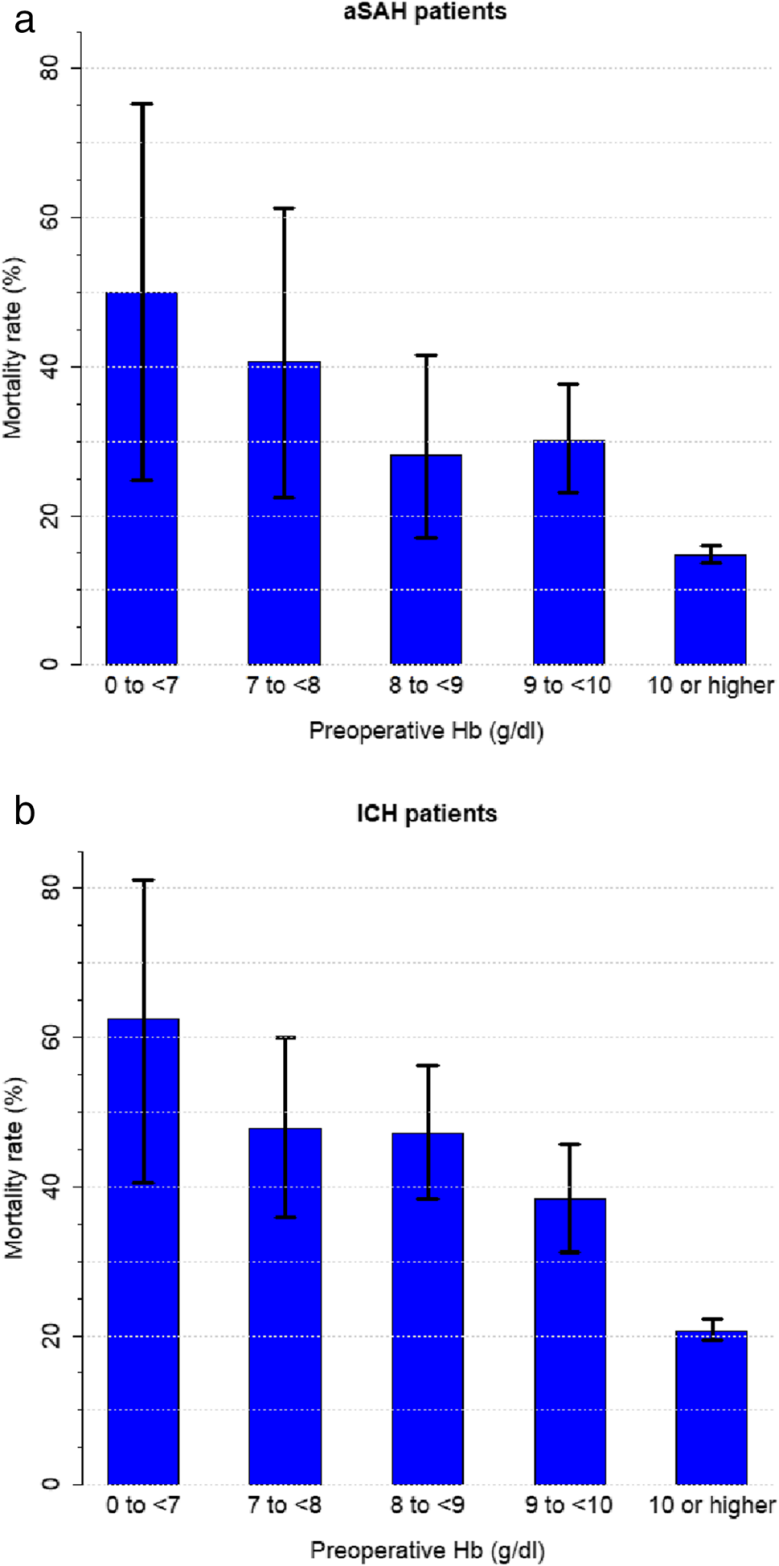
The mortality rate dependent on the preoperative Hb values for **a** Aneurysmal subarachnoid haemorrhage (aSAH) and **b** Intracerebral haemorrhage (ICH). Ninety-five percent confidence intervals (error bars) are shown

Descriptive and univariate analysis for postoperative outcomes according to preoperative anaemia are listed for both pathologies in Tables 1 and 2 and Online Resource Tables 3 and 4. Mortality was significantly higher in the presence of preoperative anaemia (22.2% versus 13.3%, *p* < 0.001 in aSAH and 31.5% versus 17.9%, *p* < 0.001 in ICH) (Table 2). Figure 2 demonstrates that an increase in the preoperative Hb values corresponds to a decrease in the mortality rate.

**Table 2:** Postoperative outcomes (LOS, mortality, complications)

Outcome	Aneurysmal subarachnoid haemorrhage (*n* = 5008)	Intracerebral haemorrhage (*n* = 4073)
Length of in-hospital stay (days)	**22.3 (± 0.3), 18.0 (9.0; 28.0)**	**23.7 (± 0.4), 16.0 (8.0; 29.0)**
In preoperative anaemic patients (days)In preoperative anaemic patients with RBC transfusion (days) In preoperative anaemic patients without RBC transfusion (days)	25.3 (± 0.8), 18.0 (9.0; 31.0) 33.4 (± 1.4), 24.0 (15.0; 41.0) 18.4 (± 0.8), 14.0 (6.8; 23.0)	25.0 (± 0.8), 15.0 (8.0; 30.0) 32.6 (± 1.4), 21.0 (10.0; 42.0) 18.7 (± 0.7), 12.0 (6.0; 23.0)
In preoperative non-anaemic patients (days)	21.0 (± 0.4), 17.0 (9.0; 26.0)	23.0 (± 0.6), 16.0 (9.0; 27.0)
Mortality	**16.4% (15.4-17.4%);** ***n*** **= 820/5008**	**23.6% (22.3-25.0%);** ***n*** **= 963/4073**
In preoperative anaemic patientsIn preoperative anaemic patients with RBC transfusion In preoperative anaemic patients without RBC transfusion	22.2% (19.9**-**24.6%); *n* = 270/1218 32.1 % (28.2 % - 36.1 %); n= 179/558 13.8 % (11.2 % - 16.7 %); n= 91/660	31.5% (29.1**-**34.0%); *n* = 448/1421 46.3 % (42.4 % - 50.3 %); n= 296/639 19.4 % (16.7 % - 22.4 %); n= 152/782
In preoperative non-anaemic patients	13.3% (12.1-14.5%); *n* = 410/3086	17.9% (16.2**-**19.6%); *n* = 367/2053
Mortality dependent on transfused RBC (units) per patient 0 RBC 1-2 RBC 3-9 RBC ≥ 10 RBC	12.4% (11.4**-**13.6%); *n* = 437/3513 19.3% (16.4**-**22.5%); *n* = 131/679 25.9% (22.5**-**29.6%); *n* = 161/621 46.7% (39.5**-**53.9%); *n* = 91/195	16.9% (15.5-18.3%); *n* = 486/2881 29.5% (25.5-33.9%); *n* = 140/474 40.9% (36.4-45.5%); *n* = 189/462 57.8% (51.5-63.9%); *n* = 148/256
Renal failure	**5.9% (5.3-6.6 %);** ***n*** **= 296/5,008**	**12.7% (11.7**-**13.8%);** ***n*** **= 517/4073**
In preoperative anaemic patientsIn preoperative anaemic patients with RBC transfusion In preoperative anaemic patients without RBC transfusion	11.4% (9.7**-**13.3 %); *n* = 139/1,218 21.0 % (17.7 % - 24.6 %); n= 117/558 3.3 % (2.1 % - 5.0 %); n= 22/660	17.8% (15.8-19.9%); *n* = 253/1421 31.8 % (28.2 % - 35.5 %); n= 203/639 6.4 % (4.8 % - 8.3 %); n= 50/782
Pulmonary embolism	**2.5% (2.0-2.9%);** ***n*** **= 123/5008**	**2.8% (2.3 % - 3.4%);** ***n*** **= 115/4073**
In preoperative anaemic patients In preoperative anaemic patients with RBC transfusion In preoperative anaemic patients without RBC transfusion	2.2% (1.5**-**3.2%); *n* = 27/1218 3.4 % (2.1 % - 5.3 %); n= 19/558 1.2 % (0.5 % - 2.4 %); n= 8/660	2.8% (2.0 % - 3.8%); *n* = 40/1421 3.9 % (2.5 % - 5.7 %); n= 25/639 1.9 % (1.1 % - 3.1 %); n= 15/782
In preoperative non-anaemic patients	2.7% (2.1**-**3.3%); *n* = 83/3086	3.1% (2.4 % - 3.9%); *n* = 63/2053
Pneumonia	**19.2% (18.2-20.4%);** ***n*** **= 964/5008**	**22.7% (21.4-24.0%);** ***n*** **= 923/4073**
In preoperative anaemic patients In preoperative anaemic patients with RBC transfusion In preoperative anaemic patients without RBC transfusion	23.4% (21.0**-**25.9%); *n* = 285/1218 37.8 % (33.8 % - 42.0 %); n= 211/558 11.2 % (8.9 % - 13.9 %); n= 74/660	22.8% (20.6**-**25.1%); *n* = 324/1421 32.6 % (28.9 % - 36.3 %); n= 208/639 14.8 % (12.4 % - 17.5 %); n= 116/782
In preoperative non-anaemic patients	17.1% (15.8**-**18.5%); *n* = 527/3086	21.5% (19.7**-**23.3%); *n* = 441/2053
Sepsis	**8.6% (7.8-9.4%);** ***n*= 429/5008**	**10.9% (10.0-11.9%);** ***n*** **= 444/4073**
In preoperative anaemic patients In preoperative anaemic patients with RBC transfusion In preoperative anaemic patients without RBC transfusion	12.8% (11.0**-**14.8%); *n* = 156/1218 22.2 % (18.8 % - 25.9 %); n= 124/558 4.8 % (3.3 % - 6.8 %); n= 32/660	15.0% (13.2**-**17.0%); *n* = 213/1421 26.4 % (23.1 % - 30.0 %); n= 169/639 5.6 % (4.1 % - 7.5 %); n= 44/782
In preoperative non-anaemic patients	6.8% (6.0**-**7.8%); *n* = 211/3086	8.6% (7.4**-**9.9%); *n* = 176/2053
Ischemic stroke	**22.4% (21.2-23.5%);** ***n*** **= 1,120/5008**	**15.5% (14.4-16.7%);** ***n*** **= 633/4073**
In preoperative anaemic patients In preoperative anaemic patients with RBC transfusion In preoperative anaemic patients without RBC transfusion	22.7% (20.3**-**25.1%); *n* = 276/1218 32.8 % (28.9 % - 36.9 %); n= 183/558 14.1 % (11.5 % - 17.0 %); n= 93/660	15.9% (14.0**-**17.9%); *n* = 226/1421 18.6 % (15.7 % - 21.9 %); n= 119/639 13.7 % (11.4 % - 16.3 %); n= 107/782
In preoperative non-anaemic patients	23.6% (22.1**-**25.1%); *n* = 728/3086	16.1% (14.6**-**17.8%); *n* = 331/2053
Myocardial infarction	**1.8% (1.4-2.2%);** ***n*** **= 90/5008**	**3.2% (2.7-3.8%);** ***n*** **= 131/4073**
In preoperative anaemic patients In preoperative anaemic patients with RBC transfusion In preoperative anaemic patients without RBC transfusion	2.6% (1.8**-**3.7%); *n* = 32/1218 4.8 % (3.2 % - 7.0 %); n= 27/558 0.8 % (0.2 % - 1.8 %); n= 5/660	3.6% (2.7**-**4.7%); *n* = 51/1421 5.9 % (4.2 % - 8.1 %); n= 38/639 1.7 % (0.9 % - 2.8 %); n= 13/782
In preoperative non-anaemic patients	1.5% (1.1**-**1.9%); *n* = 45/3086	2.9% (2.2**-**3.7%); *n* = 59/2053

Table 2 shows the postoperative outcomes depending on anaemia status. All values are represented as mean (± SE), median (IQR) or as rate (95% *CI*) and total number

Descriptive and univariate analysis revealed that preoperative anaemia resulted in significantly higher numbers of RBC units transfused, LOS, postoperative anaemia, renal failure and sepsis both for aSAH und ICH patients (Tables 1, 2, and 3, Online Resource Tables 3 and 4). Vasospasm was significantly lower in the presence of preoperative anaemia (9.3% versus 12.4%, *p* = 0.004) in aSAH patients (Table 1). Multivariate analysis showed that preoperative anaemia was an independent risk factor for increased RBC transfusion in both patients with aSAH (*p* < 0.001; *OR* = 3.25) and ICH (*p* < 0.001; *OR* = 4.16) (Tables 4 and 5). Multivariate analysis indicated preoperative anaemia was an independent risk factor for mortality (*OR* = 1.48 in aSAH patients, *OR* = 1.53 in ICH patients, both *p* < 0.001), transfused RBC units (*p* < 0.001) and postoperative anaemia (*OR* = 6.18 in aSAH patients, *OR* = 7.11 in ICH patients, *p* < 0.001). In aSAH patients, moreover, preoperative anaemia increased the risk for renal failure (*OR* = 1.61, *p* = 0.002) and LOS (+ 1.6 days, *p* = 0.03). Preoperative anaemia was an independent factor for decreased LOS in ICH (−2.5 days, *p* = 0.006). Furthermore, preoperative anaemia was an independent factor for decreased ischemic stroke (*OR* = 0.78, *p* = 0.005 in aSAH and *OR* = 0.82, *p* = 0.05 in ICH), pneumonia (*OR* = 0.78 in ICH, *p* = 0.008), pulmonary embolism (*OR* = 0.60, *p* = 0.02 in aSAH) and vasospasm (*OR* = 0.70, *p* = 0.01 in aSAH) (Tables 5 and 6).

**Table 3:** RBC-transfusion

		Aneurysmal subarachnoid haemorrhage (*n* = 5008)	Intracerebral haemorrhage (*n* = 4073)
RBC transfusion		**29.9% (28.6-31.1%);** ***n*** **= 1495/5008**	**29.3% (27.9-30.7%);** ***n*** **= 1192/4073**
In preoperative anaemic patients		45.8% (43.0**-**48.7%); *n* = 558/1218	45.0% (42.4 **-**47.6%); *n* = 639/1421
In preoperative non-anaemic patients		24.9% (23.3**-**26.4%); *n* = 767/3086	18.8% (17.1**-**20.6%); *n* = 386/2053
In patients with haemorrhagic diathesis due to coumarins In patients with haemorrhagic diathesis due to heparins In patients with haemorrhagic diathesis due to NOACs		34.9% (23.3**-**48.0%); *n* = 22/63 64.7% (46.5**-**80.3%); *n* = 22/34 41.0% (28.6**-**54.3%); *n* = 25/61	30.6% (23.9**-**38.1%); *n* = 53/173 64.3% (44.1**-**81.4%); *n* = 18/28 21.1% (15.4**-**27.8%); *n* = 38/180
In patients with Factor XIII deficiency In patients with Factor VIII deficiency		61.5% (31.6**-**86.1%); *n* = 8/13 78.3% (56.3**-**92.5%); *n* = 18/23	72.0% (50.6**-**87.9%); *n* = 18/25 73.3% (44.9**-**92.2%); *n* = 11/15
In patients with clipping In patients with coiling In patients with craniotomy		34.2% (32.0**-**36.5%); *n* = 619/1809 36.2% (33.7**-**38.8%); *n* = 503/1390 37.6% (35.6**-**39.6%); *n* = 877/2335	- - 34.7% (32.5**-**36.9%); *n* = 630/1817
RBC (units) / 1,000 patients		**1,921 (± 134)**	**2344 (± 119)**
In preoperative anaemic patients		4,272 (± 504)	4191 (± 278)
In preoperative non-anaemic patients		1,126 (± 75)	1228 (± 111)
RBC (units) / patient groups	0 RBC 1-2 RBC 3-9 RBC ≥ 10 RBC	70.1%; *n* = 3513/5008 13.6%; *n* =679/5008 12.4%; *n* = 621/5008 3.9%; *n* = 195/5008	70.7%; *n* = 2881/4073 11.6%; *n* = 474/4073 11.3%; *n* = 462/4073 6.3%; *n* = 256/4073
In preoperative anaemic patients	0 RBC 1-2 RBC 3-9 RBC ≥ 10 RBC	54.2%; *n* = 660/1218 16.7%; *n* = 204/1218 19.3%; *n* = 235/1218 9.8%; *n* = 119/1218	55.0%; *n* = 782/1421 15.1%; *n* = 214/1421 17.9%; *n* = 254/1421 12.0%; *n* = 171/1421
In preoperative non-anaemic patients	0 RBC 1-2 RBC 3-9 RBC ≥ 10 RBC	75.1%; *n* = 2319/3086 12.9%; *n* = 397/3086 10.3%; *n* = 318/3086 1.7%; *n* = 52/3086	81.2%; *n* = 1667/2053 9.2%; *n* = 188/2053 6.7%; *n* = 137/2053 3.0%; *n* = 61/2053

Table 3 shows the transfusion rates and number of RBC unitis per 1000 patients. Subanalyses were performed according to anaemia status, bleeding due to anticoagulation, factor deficiency or pro-bleeding interventions.

NOACs: Novel oral anticoagulants. All values are represented as mean (± SE) or as rate (95% *CI*) and total number

**Table 4:** Platelet, Plasma, Fibrinogen, Prothrombin complex concentrate administration

	Aneurysmal subarachnoid haemorrhage (*n* = 5008)	Intracerebral haemorrhage (*n* = 4073)
Platelet transfusion	**9.0% (8.2-9.9%);** ***n*** **= 452/5008**	**16.2% (15.1-17.4%);** ***n*** **= 660/4073**
In patients with haemorrhagic diathesis due to coumarins In patients with haemorrhagic diathesis due to heparins In patients with haemorrhagic diathesis due to NOACs In patients with Factor XIII deficiency In patients with Factor VIII deficiency	9.5% (3.6 %**-**19.6%); *n* = 6/63 44.1% (27.2**-**62.1%); *n* = 15/34 19.7% (10.6**-**31.8%); *n* = 12/61 38.5% (13.9**-**68.4%); *n* = 5/13 47.8% (26.8**-**69.4%); *n* = 11/23	11.6% (7.2**-**17.3%); *n* = 20/173 28.6% (13.2**-**48.7%); *n* = 8/28 18.9% (13.5**-**25.4%); *n* = 34/180 44.0% (24.4**-**65.1%); *n* = 11/25 26.7% (7.8**-**55.1%); *n* = 4/15
Platelet (units) / 1,000 patients	**489 (± 43)**	**984 (± 83)**
Fresh frozen plasma transfusion	**6.6% (6.0-7.4 %);** ***n*** **= 333/5008**	**9.1% (8.2-10.0%);** ***n*** **= 369/4073**
In patients with haemorrhagic diathesis due to coumarins In patients with haemorrhagic diathesis due to heparins In patients with haemorrhagic diathesis due to NOACs In patients with Factor XIII deficiency In patients with Factor VIII deficiency	12.7% (5.6**-**23.5 %); *n* = 8/63 41.2% (24.6**-**59.3 %); *n* = 14/34 14.8% (7.0**-**26.2 %); *n* = 9/61 15.4% (1.9**-**45.4 %); *n* = 2/13 47.8% (26.8**-**69.4 %); *n* = 11/23	8.7% (4.9**-**13.9%); *n* = 15/173 42.9% (24.5**-**62.8%); *n* = 12/28 10.0% (6.0**-**15.3%); *n* = 18/180 20.0% (6.8**-**40.7%); *n* = 5/25 40.0% (16.3**-**67.7%); *n* = 6/15
Fresh frozen plasma (units) / 1,000 patients	**596 (± 53)**	**989 (± 94)**
Fibrinogen administration	**3.8% (3.3-4.4%);** ***n*** **= 190/5008**	**6.4% (5.6-7.2%);** ***n*** **= 259/4073**
In patients with haemorrhagic diathesis due to coumarins In patients with haemorrhagic diathesis due to heparins In patients with haemorrhagic diathesis due to NOACs In patients with Factor XIII deficiency In patients with Factor VIII deficiency	4.8% (1.0**-**13.3%); *n* = 3/63 17.6% (6.8**-**34.5%); *n* = 6/34 9.8% (3.7**-**20.2%); *n* = 6/61 15.4% (1.9**-**45.4%); *n* = 2/13 30.4% (13.2**-**52.9%); *n* = 7/23	7.5% (4.1**-**12.5%); *n* = 13/173 21.4% (8.3**-**41.0%); *n* = 6/28 8.9% (5.2**-**14.0%); *n* = 16/180 24.0% (9.4**-**45.1%); *n* = 6/25 0.0% (0.0**-**21.8%); *n* = 0/15
Fibrinogen (g) / 1,000 patients	**202 (± 30)**	**484 (± 76)**
Prothrombin complex concentrate administration	**8.7% (7.9-9.5%);** ***n*** **= 435/5008**	**20.9% (19.7-22.2%);** ***n*** **= 851/4,073**
In patients with haemorrhagic diathesis due to coumarins In patients with haemorrhagic diathesis due to heparins In patients with haemorrhagic diathesis due to NOACs In patients with Factor XIII deficiency In patients with Factor VIII deficiency	77.8% (65.5**-**87.3%); *n* = 49/63 38.2% (22.2**-**56.4%); *n* = 13/34 39.3% (27.1**-**52.7%); *n* = 24/61 23.1% (5.0**-**53.8%); *n* = 3/13 34.8% (16.4**-**57.3%); *n* = 8/23	74.0% (66.8**-**80.4%); *n* = 128/173 28.6% (13.2**-**48.7%); *n* = 8/28 55.0% (47.4**-**62.4%); *n* = 99/180 32.0% (14.9**-**53.5%); *n* = 8/25 6.7% (0.2**-**31.9%); *n* = 1/15
Prothrombin complex concentrate (IE)/1000 patients	**320,022 (± 30,449)**	**796,704 (± 42,540)**

Tables 4 shows the administration rates for platelet, plasma, fibrinogen and prothrombin complex concentrate administration in aSAH and ICH patients. Sub-analyses were performed for factor deficiency syndromes and anticoagulants. All values are represented as mean (± SE) or as rate (95% CI) and total number

**Table 5:** Multivariate regression analysis: risk factors on postoperative outcomes for aSAH patients

Outcomes: →	Vasospasm	RBC transfusion	Mortality	LOS	RBC units/1000 patients	Postoperative anaemia	Hospital acquired anaemia	Myocardial infarction	Ischaemic stroke	Renal failure	Sepsis	Pneumonia	Pulmonary embolism
Risk factors↓													
Age (per increase of 10 years)	*P* < 0.001; *OR* = 0.82 (0.75-0.89)	*P* < 0.001; *OR* = 1.11 (1.06-1.17)	*P* < 0.001; *OR* = 1.27 (1.19-1.36)	n.s.	n.s.	*P* < 0.001; *OR* = 1.14 (1.07-1.21)	*P* = 0.001; *OR* = 1.12 (1.04-1.20)	*P* < 0.001; *OR* = 1.32 (1.13-1.54)	n.s. *P* = 0.06; *OR* = 1.06 (1.00-1.12)	*P* = 0.007; *OR* = 1.15 (1.04-1.27)	n.s.	*P* < 0.001; *OR* = 1.16 (1.09-1.22)	n.s.
. Gender (female vs male)	*P* = 0.02; *OR* = 1.32 (1.04-1.68)	P < 0.001; *OR* = 1.51 (1.30-1.75)	*P* = 0.002; *OR* = 0.76 (0.63-0.90)	*P* < 0.001; −3.0 ± 0.6 day	*P* = 0.053; -604 ± 312	n.s.	n.s.	*P* = 0.01; *OR* = 0.58 (0.38-0.89)	*P* = 0.01; *OR* = 0.82 (0.70-0.96)	*P* < 0.001; *OR* = 0.50 (0.38-0.67)	*P* < 0.001; *OR* = 0.50 (0.40-0.62)	*P* < 0.001; *OR* = 0.58 (0.50-0.68)	*P* = 0.04; *OR* = 0.67 (0.46-0.99)
Preoperative anaemia	*P* =0.01; *OR* = 0.70 (0.53-0.92)	*P* < 0.001; *OR* = 3.25 (2.79-3.79)	*P* < 0.001; *OR* = 1.48 (1.23-1.79)	*P* = 0.03; 1.6 ± 0.7 day	*P* < 0.001; 3033 ± 339	*P* < 0.001; *OR* = 6.18 (4.61-8.28)	-	n.s.	. *P* = 0.005; *OR* = 0.78 (0.65-0.93)	*P* = 0.002; *OR* = 1.61 (1.20-2.16)	n.s.	n.s.	*P* = 0.02; *OR* = 0.60 (0.38-0.94)
Clipping	*P* < 0.001; *OR* = 2.10 (1.60-2.76)	n.s.	*P* < 0.001; OR = 0.40 (0.31-0.52)	n.s.	*P* = 0.035; −679 ± 322	n.s.	*P* = 0.02; *OR* = 1.41 (1.06-1.87)	*P* < 0.001; *OR* = 4.18 (2.71-6.45)	n.s.	*P* < 0.001; *OR* = 0.26 (0.15-0.42)	n.s.	n.s.	n.s.
Coiling	*P* < 0.001; *OR* = 16.23 (12.41-21.23)	*P* < 0.001; *OR* = 1.63 (1.37-1.94)	*P* = 0.04; *OR* = 0.80 (0.65-0.99)	*P* = 0.006; 2.2 ± 0.8 days	n.s.	*P* < 0.001; *OR* = 1.86 (1.46-2.36)	*P* < 0.001; *OR* = 2.04 (1.58-2.62)	n.s.	*P* < 0.001; *OR* = 1.84 (1.54-2.21)	*P* < 0.001; *OR* = 0.27 (0.18-0.40)	n.s.	*P* < 0.001; *OR* = 1.49 (1.24-1.78)	n.s.
Craniotomy	n.s.	*P* < 0.001; *OR* = 2.30 (1.97-2.69)	*P* < 0.001; *OR* = 1.56 (1.24-1.98)	*P* = 0.01; −1.7 ± 0.7 days	n.s.	*P* < 0.001; *OR* = 2.25 (1.87-2.72)	*P* < 0.001; *OR* = 1.89 (1.45-2.47)	n.s.	*P* < 0.001; *OR* = 1.43 (1.21-1.69)	*P* = 0.002; *OR* = 0.53 (0.36-0.79)	*P* < 0.001; *OR* = 0.54 (0.43-0.67)	n.s.	n.s.
Vasospasm	-	P < 0.001; *OR* = 2.13 (1.69-2.69)	n.s.	*P* < 0.001; 6.1 ± 1.1	n.s.	*P* = 0.004; *OR* = 1.67 (1.18-2.36)	*P* = 0.006; *OR* = 1.66 (1.15-2.39)	n.s.	*P* < 0.001; *OR* = 1.76 (1.38-2.25)	n.s.	n.s.	*P* = 0.001; *OR* = 1.45 (1.16-1.81)	n.s.
ICH	n.s.	*P* < 0.001; *OR* = 2.16 (1.84-2.55)	*P* < 0.001; *OR* = 2.23 (1.83-2.71)	n.s.	n.s.	*P* < 0.001; *OR* = 1.56 (1.23-1.97)	*P* < 0.001; *OR* = 1.69 (1.31-2.18)	n.s.	*P* = 0.05; *OR* = 1.19 (1.00-1.42)	n.s.	n.s.	*P* < 0.001; *OR* = 1.56 (1.30-1.86)	n.s.
RBC transfusion	. *P* < 0.001; *OR* = 2.47 (1.94-3.15)	-	*P* < 0.001; *OR* = 2.30 (1.91-2.78)	*P* < 0.001; 13.7 ± 0.7	-	*P* < 0.001; *OR* = 4.70 (3.51-6.30)	*P* < 0.001; *OR* = 7.15 (4.95-10.32)	*P* < 0.001; *OR* = 0.25 (0.13-0.48)	*P* < 0.001; *OR* = 3.19 (2.70-3.76)	*P* < 0.001; *OR* = 12.08 (8.65-16.87)	*P* < 0.001; *OR* = 9.43 (7.43-11.96)	*P* < 0.001; *OR* = 5.42 (4.60-6.38)	*P* < 0.001; *OR* = 3.67 (2.46-5.45)

Table 5 shows the results of the multivariate regression analysis to investigate risk factors for postoperative outcomes in aSAH patients. The vertical column lists the risk factors and the horizontal column lists the corresponding outcomes. All values are represented either as Odds ratio (with 95% *CI*) for binary endpoints or as difference in mean ± standard error of mean (SE) for continuous endpoints. Non-significant results and p-values > 0.10 are marked with n.s.

**Table 6:** Multivariate regression analysis: Independent risk factors on postoperative outcomes for ICH patients

Outcomes												
	RBC transfusion	Mortality	LOS	RBC units/1000 patients	Postoperative anaemia	Hospital acquired anaemia	Myocardial infarction	Ischaemic stroke	Renal failure	Sepsis	Pneumonia	Pulmonary embolism
Risk factors												
Age (per increase of 10 years)	*P* < 0.001; *OR* = 0.89 (0.84-0.94)	*P* < 0.001; *OR* = 1.18 (1.11-1.25)	*P* < 0.001; −1.0 ± 0.3 days	*P* < 0.001; −661 ± 90	*P* = 0.006; *OR* = 1.10 (0.60-1.65)	*P* = 0.004; *OR* = 1.11 (1.04-1.20)	*P* = 0.01; *OR* = 1.18 (1.04-1.36)	n.s.	n.s.	*P* < 0.001; *OR* = 0.85 (0.79-0.91)	n.s.	n.s.
Gender (female vs male)	*P* = 0.003; *OR* = 1.28 (1.09-1.50)	n.s.	*P* < 0.001; −3.4 ± 0.9 days	n.s.	*P* < 0.001; *OR* = 0.63 (0.52-0.77)	*P* < 0.001; *OR* = 0.68 (0.55-0.84)	*P* < 0.001; *OR* = 0.44 (0.30-0.66)	n.s.	*P* < 0.001; *OR* = 0.57 (0.46-0.70)	*P* < 0.001; *OR* = 0.67 (0.54-0.84)	*P* < 0.001; *OR* = 0.58 (0.49-0.70)	n.s.
Preoperative Anaemia	*P* < 0.001; *OR* = 4.16 (3.54-4.89)	*P* < 0.001; *OR* = 1.53 (1.29-1.82)	*P* = 0.006; −2.5 ± 0.9 days	*P* < 0.001; 2881 ± 264	*P* < 0.001; *OR* = 7.11 (5.30-9.54)	-	n.s.	*P* = 0.05; *OR* = 0.82 (0.67-1.00)	n.s.	n.s.	*P* = 0.008; *OR* = 0.78 (0.65-0.94)	n.s.
Craniotomy	*P* < 0.001; *OR* = 1.78 (1.49-2.08)	*P* = 0.01; *OR* = 0.80 (0.67-0.95)	*P* < 0.001; −5.3 ± 0.9 days	*P* < 0.001; −1263 ± 268	*P* < 0.001; *OR* = 1.76 (1.43-2.16)	*P* < 0.001; *OR* = 1.79 (1.45-2.22)	*P* = 0.008; *OR* = 0.60 (0.41-0.88)	*P* = 0.005; OR = 1.31 (1.08-1.60)	*P* < 0.001; *OR* = 0.37 (0.30-0.46)	*P* < 0.001; *OR* = 0.34 (0.27-0.43)	*P* < 0.001; *OR* = 0.74 (0.62-0.88)	n.s.
RBC transfusion	-	*P* < 0.001; *OR* = 3.59 (3.00-4.30)	*P* < 0.001; +17.7 ± 1.0 days	-	*P* < 0.001; *OR* = 7.34 (4.96-10.87)	*P* < 0.001; *OR* = 8.14 (5.16-12.86)	*P* < 0.001; *OR* = 3.57 (2.48-5.15)	*P* < 0.001; *OR* = 2.13 (1.73-2.62)	*P* < 0.001; *OR* = 9.45 (7.60-11.75)	*P* < 0.001; *OR* = 7.92 (6.27-9.99)	*P* < 0.001; *OR* = 3.29 (2.73-3.98)	*P* < 0.001; *OR* = 2.12 (1.46-3.09)

Table 6 shows the results of the multivariate regression analysis to investigate risk factors for postoperative outcomes in ICH patients. The vertical column lists the risk factors and the horizontal column lists the corresponding outcomes. All values are represented either as Odds ratio (with 95% *CI*) for binary endpoints or as difference in mean ± standard error of mean (SE) for continuous endpoints. Non-significant results and p-values > 0.10 are marked with n.s.

### RBC transfusion

**Fig. 3:**
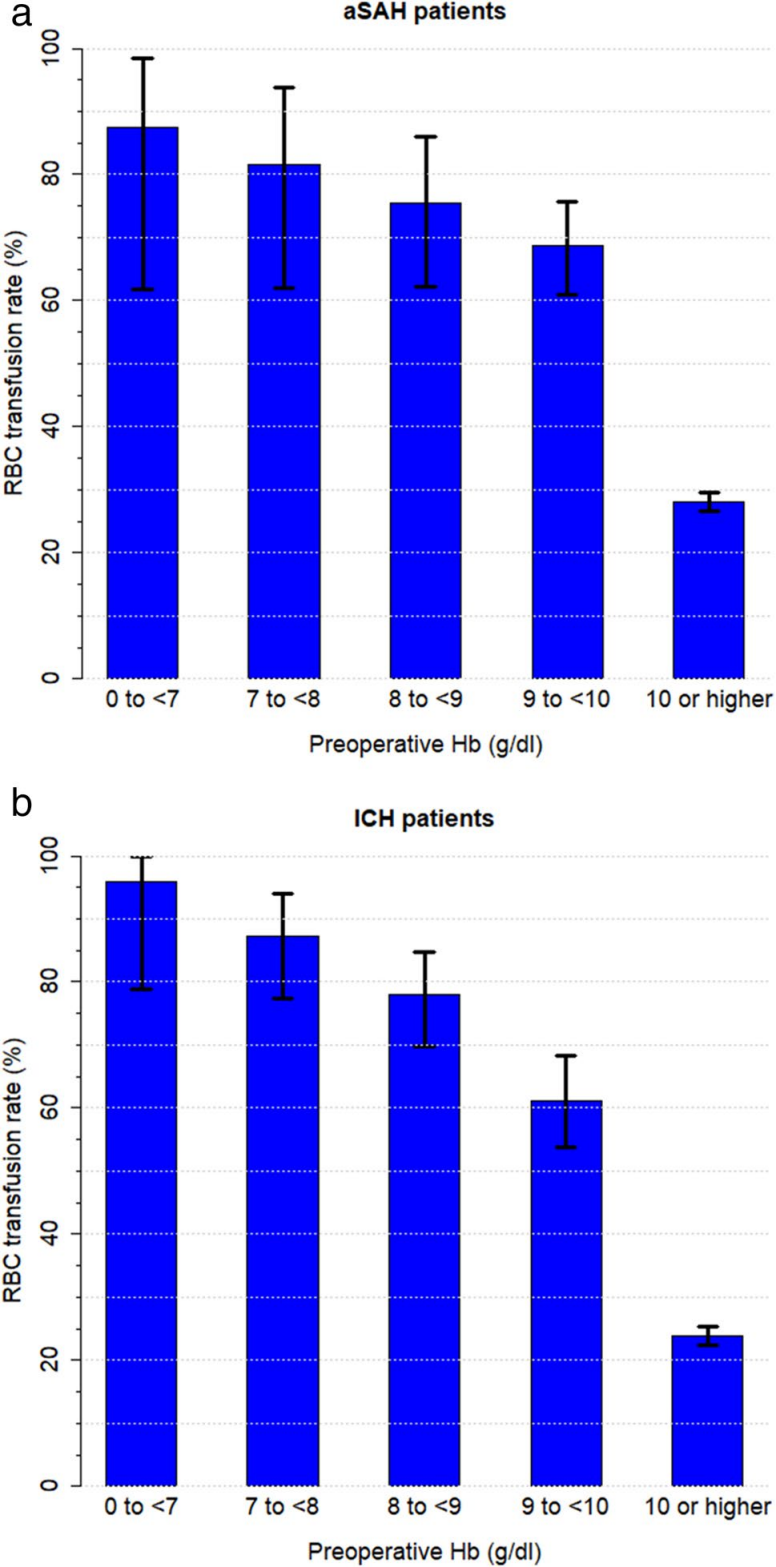
The RBC transfusion rate dependent on the preoperative Hb values for **a** Aneurysmal subarachnoid haemorrhage (aSAH) and **b** Intracerebral haemorrhage (ICH). Ninety-five percent confidence intervals (error bars) are shown

RBC transfusion rates were higher in the presence of preoperative anaemia in both the aSAH group (45.8% vs 24.9%, *p* < 0.001) and the ICH group (45.0% vs 18.8%, *p* < 0.001), (Table 3 and Online Resource Tables 3 and 4). Figure 3 demonstrates that a constant increase in the preoperative Hb values corresponds to a constant decrease in the RBC transfusion rate. Preoperative anaemic patients were significantly more likely to receive RBC transfusions than non-anaemic patients (24.9% vs. 45.8%, *p* < 0.001 in aSAH and 18.8% vs. 45.0%, *p* < 0.001 in ICH) (Table 3 and Online Resource Tables 3 and 4). In the additional descriptive analysis, transfusion rates for RBC, plasma and clotting products were higher when haemorrhagic diatheses due to coumarins, heparins and novel oral anticoagulants (NOACs), as well as factor XIII and factor VIII deficiency, were present (Table 3 and 4). Mortality rates were higher when more RBC units were required (Table 2).

Multivariate analysis revealed that RBC transfusion was an independent (all *p* < 0.001) risk factor for increased mortality (*OR* = 3.59 in ICH, *OR* = 2.30 in aSAH), LOS (+ 17.7 days in ICH, + 13.7 days in aSAH), ischaemic stroke, renal failure, sepsis, pneumonia and pulmonary embolism in aSAH and ICH patients and for vasospasm (*OR* = 2.47) in aSAH patients (Table 5 and 6).

### Interventions

In the univariate and descriptive analysis, RBC transfusion rates were significantly (*p* < 0.001) higher in the presence of interventions (Table 2 and Online Resource Tables 3 and 4). In the multivariate analysis, clipping was an independent factor for significantly lesser RBC units transfused (−679 units/1000 patients, *p* = 0.035) in aSAH patients. Coiling (*OR* = 1.63, *p* < 0.001) and craniotomy (*OR* = 2.30, *p* < 0.001) were independent risk factors for significantly higher RBC transfusion rates in aSAH patients. Craniotomy was independently associated with significantly higher RBC transfusion rates (*OR* = 1.78, *p* < 0.001 in ICH and *OR* = 2.30, *p* < 0.001 in aSAH) and RBC units transfused (+ 1263 units/1000 patients, *p* < 0.001 in ICH) (Table 5 and 6)

### Vasospasm

The proportion of preoperative anaemia was significantly lower (*p* = 0.004) in the vasospasm (22.8%) group than in the non-vasospasm group (29.0%). The RBC transfusion rate was significantly higher (*p* < 0.001) in the vasospasm (40.9%) than in the non-vasospasm group (28.1%) (Online Resource 5). In the multivariate analysis, vasospasm was an independent risk factor for RBC transfusion (*OR* = 2.13, *p* < 0.001), postoperative anaemia (*OR* = 1.67, *p* = 0.004), prolonged LOS (+ 6.1 days, *p* < 0.001), pneumonia (*OR* = 1.45, *p* < 0.001) and ischemic stroke (*OR* = 1.45, *p* = 0.001) (Tables 3 and 4).

## Discussion

The data revealed in both aSAH and ICH patients that preoperative anaemia is associated with a higher RBC transfusion rate, increased postoperative in-hospital mortality and increased complication rates. These findings align with the study results for other patient cohorts. Thus, in neurosurgical patients, preoperative anaemia has been shown to be an independent risk factor for postoperative mortality and increased risk of RBC transfusion [24]. Anaemia is common in aSAH patients [8, 14, 30, 33] and in ICH patients [16, 17]. In this study, the prevalence of preoperative anaemia in both groups (aSAH 28.3% and ICH 40.9%) was higher than described in previous publications (aSAH 5.5% and ICH 24.1-25.8%) [8, 16, 17]. One explanation for this could be that the database only includes cohorts of patients who underwent surgery or other interventions (e.g., coiling) during a hospital stay, so that selection bias cannot be ruled out.

The physiological and pathophysiological impact of anaemia in patients with aSAH and ICH is multifactorial. The supply of oxygen to the brain depends on several variables. Cerebral oxygen availability (DO_2_) is the product of cerebral blood flow (CBF) and arterial oxygen content (CaO_2_): DO_2_ = CBF × CaO2 [19]. The oxygen content (CaO_2_) itself is represented by the formula CaO_2_ = (1.31 × Hb × SaO_2_ × 0.01) + (0.0225 × PaO_2_) and thus depends on Hb levels, arterial oxygen saturation (SaO_2_) and arterial oxygen pressure (PaO_2_) [7]. The formula demonstrates that apart from an increase in SaO_2_, the most significant factor for optimising the DO_2_ to the target cell is the Hb value; thus, the need arises to consider ways of increasing the Hb value through various measures (such as anaemia management or transfusion in an emergency). In a healthy brain, a progressive decrease in Hb is compensated for by vasodilation, resulting in increased CBF and a constant cerebral oxygen supply DO_2_. When Hb falls below 5-6 g/dL, DO_2_ decreases and no further vasodilation can occur and maximum CBF levels are reached [19]. We observed that the vasospasm rate was significantly lower with preoperative anaemia. It is possible, that the Hb value influences only patients’ outcomes after cerebral vasospasm and not the probability of a cerebral vasospasm event itself. The multivariate analysis also revealed a significant association of RBC transfusion (*OR* = 2.47, *p* < 0.001) with vasospasm. This finding underlines the need for risk assessment prior to transfusion and additional prospective studies on this topic. Scholars have long debated whether elevating the haemoglobin levels in SAH patients with vasospasm and thus avoiding anaemia is beneficial [15, 18, 29]. In general, based on CONSCIOUS-3, the role of vasospasm on delayed cerebral ischemia should be considered with caution, where clazosentan was shown to significantly reduce postaSAH vasospasm, but neither dose improved outcome [20]. Further studies are needed to prove the potential beneficial effects of RBC transfusion on anaemic aSAH patients suffering from cerebral vasospasm. In the field of critical care, there is a growing evidence that strict transfusion limits remain best practice for the vast majority of cases, due to limited adverse effects, comparable or better clinical outcomes and economic aspects [28]. Thus, a restrictive threshold for RBC transfusions (Hb < 7 g/dl) is still recommended in both critically ill and clinically stable ICU patients [23]. Similar pathophysiological considerations are known in patients with acute myocardial infarction, as a recent study demonstrated that a restrictive transfusion strategy resulted in less major adverse cardiac events after 30 days (11.0% in the restrictive and 14.0% in the liberal group) [6]. In the retrospective study by English et al., only 20% of patients with aSAH received RBC transfusions, mostly in the presence of significant anaemia (Hb < 8 g/dl), and this was not associated with worse outcomes [8]. However, Dhar et al. demonstrated that RBC transfusion in aSAH patients improved cerebral oxygenation both globally and particularly in the vulnerable brain regions and thus may potentially minimise the risk for delayed cerebral ischaemia. The study analysed the outcomes over a wide range of haemoglobin levels and suggests that restrictive transfusion practice may not be appropriate in this vulnerable population [5]. Naidech et al. demonstrated no difference in outcomes in SAH for Hb 10.0 versus 11.5 g/dL. Here, however, the difference between the groups is rather minor and well-above general limits for transfusions [25]. The answer to the question of the role of treatment of anaemia with red blood cell transfusion could be provided by the still ongoing SAHaRA trial [9]. In our analysis, mortality was increased considerably when more transfusions were given, which is also in line with the results from Ceanga et al. [3].

Although preoperative diagnosis and treatment of anaemia can only be implemented to a limited extent in acute situations of ICH and aSAH, the present data underlines primarily the importance of general anaemia vigilance and treatment (as ICH and aSAH occur sudden and without time frame for treatment), and secondarily, anaemia treatment becomes important in the context of peri-/postoperative care. To identify and manage anaemia at an early stage, a multimodal therapy using patient blood management (PBM) has been developed. PBM is an evidence-based, patient-centred and multidisciplinary approach to minimise anaemia-associated risks, unnecessary blood loss and transfusions in patients undergoing surgery [1]. For this purpose, measures have been implemented to reduce preoperative anaemia, minimise iatrogenic blood loss and optimise patient-specific anaemia tolerance [11]. If iron deficiency is identified in the absence of infection, iron supplementation and erythropoiesis-stimulating agents can be considered [13]. Measures to reduce intraoperative blood loss and optimise coagulopathy should be implemented. This includes the following measures (also in neurosurgery): Treatment of coagulopathy should be based on a fixed algorithm. The content of the coagulation algorithm should be the maintenance of basic conditions for haemostasis (body temperature > 36 °C, ionised calcium > 1.1 mmol/ L, pH > 7.2) or point-of-care diagnostics. The prevalence of bleeding due to anticoagulation was low in our analysis but point of care technology provides information on coagulation dysfunction and the use of anticoagulation, including NOACs. The use of an antifibrinolytic is safe and recommended [32]. Blood sample collections should be reduced to the absolute necessary numbers, blood sample collection tubes should draw as little blood volume as possible, and return systems for blood sample collections should be established. Washed cell salvage—the collection, washing and retransfusion of a patient’s own wound blood—can help to reduce the need for blood from other sources [21, 26].

### Limitations

Although studies with routine data have several important advantages over traditional clinical trials (such as a larger number of cases with fewer personnel, time and cost requirements) and are therefore becoming increasingly popular as an alternative in the age of advancing digitalisation, they naturally also have some disadvantages. This study is based on routine data of hospital information systems. Data quantity and quality varied between hospitals. In addition, routine data may have some other limitations, in general, such as missing data or incorrect coding techniques. Since ICD and OPS codes are billing-related, they may be biased.

Furthermore, there is no information on the exact time of occurrence and duration of complications and perioperative interventions (including blood transfusions), so an association does not necessarily indicate causality, nor is it possible to show the direction of causality. For this reason, we report associations rather than causalities of the factors.

Missing information on neurological status, resuscitation and intercurrent diseases cannot be obtained from the register, so that a limitation in the analysis of associations with anaemia and transfusion is possible here. The analysis could not consider the influence of a potential — and already locally available — anaemia therapy. The Hunt and Hess scale for aSAH, which measures the severity of the aSAH, is not documented in ICD-10 codes; therefore, a severity-adapted evaluation was not possible. In neurosurgical therapy, patients are often transferred to a rehabilitation intensive care unit or back to the referring intensive care unit shortly after treatment; this leads to a possible bias in endpoints (e.g., especially for LOS). This is a retrospective analysis of prospectively collected registry data; limitations of a retrospective analysis cannot be avoided.

## Conclusions

Preoperative anaemia is associated with increased RBC transfusion rates, in-hospital mortality, and postoperative complications in patients with aSAH and ICH. Prospective multicentre studies with tailored data on the therapy of anaemia, the optimal haemoglobin value and transfusion strategy, both for aSAH and ICH patients, are urgently needed.

## Supplementary Information

ESM 1 (DOCX 42 kb)

## Abbreviations

aSAH: aneurysmal subarachnoid haemorrhage
CI: confidence interval
Hb: haemoglobin
ICD: International Classification of Disease
ICH: intracerebral haemorrhage
IQR: interquartile range
LOS: Length of in-hospital stay
NOAC: novel oral anticoagulants
OPS: Operation and Procedure Classification
OR: odds ratio
RBC: red blood cells
SE: standard error
