# Supplementary material for: Preoperative anaemia and red blood cell transfusion in patients with aneurysmal subarachnoid and intracerebral haemorrhage — a multicentre subanalysis of the German PBM Network Registry
Source: Acta Neurochir (Wien). 2022 Feb 26;164(4):985–99. doi: 10.1007/s00701-022-05144-7 (PMC8967742; doi:10.1007/s00701-022-05144-7)
Supplement: Supplementary file 1 — (DOCX 42 kb) [file 701_2022_5144_MOESM1_ESM.docx]

**Online Resource 1) ICD and OPS Codes for complications and interventions**

| **Bleedings** |  |
| --- | --- |
| Intracerebral haemorrhage | I61 (I61.0-7 and I61.9) |
| Aneurysmal subarachnoid haemorrhage | I60 (I60.0-9), I67.0, I67.1 |
| Traumatic subarachnoid haemorrhage (for exclusion) | S06.6 |
| **Neoplasm** |  |
| Glioblastoma (for exclusion) | C71 |
| Meningeoma (for exclusion) | C70 |
| **Vasospasm** |  |
| Vasospasm (diagnosis) | I67.80 |
| Spasmolysis in cerebrovascular vasospasm (intervention against vasospasm) | 8-83c.6, 8-83c.7 |
| **Haemorrhagic diathesis and factor deficiency** |  |
| Haemorrhagic diathesis due to coumarins | D68.33 |
| Haemorrhagic diathesis due to heparins | D68.34 |
| Haemorrhagic diathesis due NOACs | D68.35 |
| Factor XIII deficiency | D68.26 |
| Factor VIII deficiency | D66, D68.00, D68.01, D68.09 |
| **Extracorporeal devices** |  |
| Left Ventricular Assist Device | 5-376 |
| Extracorporeal membrane oxygenation | 8-852.0 |
| Extracorporeal life support | 8-852.3, 8-852.6 |
| **Interventions** |  |
| Clipping | 5-025, 5-026 |
| Coiling | 8-836.00, 8-836.01, 8-836.0h, 8-836.9, 8-836.b, 8-836.c, 8-836.k, 8-836.m |
| Craniotomy | 5-010.0, 5-010.1, 5-010.3, 5-012.0 |
| **Complications** |  |
| Renal failure | N17, N17.0, N17.1, N17.2, N17.8, N17.9, N19, N99.0 |
| Pneumonia | J12, J12.0, J12.1, J12.2, J12.3, J12.8, J12.9, J13, J14, J15, J15.0, J15.1, J15.2, J15.3, J15.4, J15.5, J15.6, J15.7, J15.8, J15.9, J16 J16.0, J16.8, J18, J18.0, J18.1, J18.2, J18.8, J18.9 |
| Sepsis | A40, A40.0, A40.1, A40.2, A40.3, A40.8, A40.9, A41, A41.0, A41.1, A41.2, A41.3, A41.4, A41.51, A41.52, A41.8, A41.9, A42.7 B00.7, B37.7 |
| Ischemic stroke | I63, I63.0, I63.1, I63.2, I63.3, I63.4, I63.5, I63.6, I63.8, I63.9, I64 |
| Myocardial infarction | I21, I21.0, I21.1, I21.2, I21.3, I21.4, I21.9, I22, I22.0, I22.1, I22.8, I22.9 |
| Pulmonary embolism | I26.0, I26.9 |

Online Resource 1 shows the ICD and OPS codes used to define the groups and outcomes.

ICD code: The International Classification of Disease Code (ICD) is the official classification for coding medical diseases.

OPS code: The Operation and Procedure Code (OPS) is the official classification for coding operations, procedures and general medical measures.

**Online Resource 2) Surgical procedures of the underlying registry in aSAH and ICH patients**

| **Surgery groups** | **OPS Codes** | **aSAH (100.0 %; n= 5,008)** | **ICH (100.0 %; n=4,073)** |
| --- | --- | --- | --- |
| Neurosurgery | 5-01 to 5-05 | 57.4 %; n= 2,876 | 49.9 %; n= 2,034 |
| Neurosurgery + 2^nd^ Surgery Group | 5-01 to 5-05 +5-06 to 5-99 | 27.5 %; n=1,390 | 27.0 %; n= 1,098 |
| Mixed group excluding Neurosurgery | 5-06 to 5-99 | 3.5 %; n= 177 | 6.2 %; n= 253 |
| Dermatology, ophthalmology | 5-08 to 5-16, 5-89 to 5-99 | 2.5 %; n= 127 | 1.8 %; n= 75 |
| Otorhinolaryngology | 5-18 to 5-31 | 1.9 %; n= 93 | 3.5 %; n= 141 |
| Thoracic surgery | 5-32 to 5-34 | 0.2 %; n= 9 | 0.3 %; n= 11 |
| Cardiac surgery | 5-35 to 5-37 | 1.2 %; n= 60 | 1.9 %; n= 76 |
| Vascular surgery | 5-38 to 5-39 | 1.3 %; n= 65 | 1.9 %; n= 76 |
| Visceral and endocrine surgery | 5-06 to 5-07, 5-42 to 5-45 | 2.0 %; n= 102 | 5.0 %; n= 205 |
| Urology | 5-55 to 5-64 | 0.2 %; n= 12 | 0.7 %; n= 27 |
| Gynaecology | 5-65 to 5-71, 5-87 to 5-88 | 0.1 %; n= 4 | 0.1 %; n= 3 |
| Obstetric | 5-72 to 5-75 | 0.3 %; n= 17 | 0.2 %; n= 7 |
| Oral-maxillofacial surgery | 5-76 to 5-77 | 0.3 %; n= 17 | 0.1 %; n= 6 |
| Trauma and orthopaedic surgery | 5-78 to 5-86 | 1.1 %; n= 55 | 1.3 %; n= 53 |
| Other (surgery of the hematopoietic- and lymphatic system) | 5-40 to 5-41 | 0.1 %; n= 4 | 0.2 %; n= 8 |

OPS code: The Operation and Procedure Code is the official classification for coding operations, procedures and general medical measures.

**Online Resource 3) Univariate analysis for risk factors on postoperative outcomes for Aneurysmal subarachnoid haemorrhage (aSAH) patients**

| **Outcomes: →** | **Vasospasm** | **RBC**  **transfusion** | **Mortality** | **LOS** | **RBC units** | **Postoperative anaemia** | **Hospital acquired anaemia** | **Preoperative anaemia** | **Myocardial infarction** | **Ischaemic**  **stroke** | | **Renal**  **failure** | **Sepsis** | **Pneumonia** | **Pulmonary**  **embolism** |
| --- | --- | --- | --- | --- | --- | --- | --- | --- | --- | --- | --- | --- | --- | --- | --- |
| **Risk factors: ↓** |  |  |  |  |  |  |  |  |  |  |  |  |  |  |  |
| **Age** | n.s. | n.s. (0.07) | <0.001 | 0.05 | n.s. | <0.001 | n.s. | n.s. | n.s. | n.s. | <0.001 | | <0.001 | n.s. (0.10) | n.s. |
| **Gender** | 0.001 | <0.001 | 0.002 | n.s. | <0.001 | n.s. | n | n.s. | 0.03 | n.s. | <0.01 | | <0.001 | <0.001 | 0.05 |
| **Preoperative anaemia** | 0.004 | <0.001 | <0.001 | 0.03 | <0.001 | <0.001 | - | - | 0.01 | n.s. | <0.001 | | <0.001 | <0.001 | n.s. |
| **Clipping** | 0.01 | <0.001 | <0.001 | 0.02 | <0.001 | 0.03 | <0.001 | - | <0.001 | n.s. | <0.001 | | <0.001 | 0.03 | n.s. (0.08) |
| **Coiling** | <0.001 | <0.001 | n.s. | <0.001 | <0.001 | <0.001 | <0.001 | - | n.s. | <0.001 | <0.001 | | n.s. | <0.001 | n.s. |
| **Craniotomy** | n.s. | <0.001 | n.s. | 0.04 | <0.001 | <0.001 | <0.001 | - | <0.001 | <0.001 | <0.001 | | <0.001 | n.s. | n.s. (0.09) |
| **Vasospasm** | - | <0.001 | n.s. | <0.001 | <0.001 | <0.001 | <0.001 | - | n.s. | <0.001 | n.s. | | <0.001 | <0.001 | n.s. |
| **Additional ICH** | n.s. | <0.001 | <0.001 | <0.001 | <0.001 | <0.001 | <0.001 | - | n.s. | <0.001 | n.s. | | n.s. | <0.001 | 0.02 |
| **RBC Transfusion** | <0.001 | - | <0.001 | <0.001 | - | <0.001 | <0.001 | - | <0.001 | <0.001 | <0.001 | | <0.001 | <0.001 | <0.001 |
| **RBC units** | 0.004 | - | <0.001 | <0.001 | - | <0.001 | <0.001 | - | <0.001 | <0.001 | <0.001 | | <0.001 | <0.001 | <0.001 |

Online Resource 3 shows the results of the univariate analysis for risk factors on postoperative outcomes for aneurysmal subarachnoid haemorrhage (aSAH) patients; n.s.= not significant (with p-values ≤ 0.10 still explicitly displayed); ICH: Intracerebral haemorrhage, LOS: Length of in-hospital stay

**Online Resource 4) Univariate analysis for risk factors on postoperative outcomes for Intracerebral haemorrhage (ICH) patients**

| **Outcomes: →** | **RBC**  **transfusion** | **Mortality** | **LOS** | **RBC**  **units** | **Postoperative**  **anaemia** | **Hospital acquired**  **anaemia** | **Preoperative**  **anaemia** | **Myocardial**  **infarction** | **Ischaemic**  **stroke** | **Renal**  **failure** | **Sepsis** | **Pneumonia** | **Pulmonary**  **embolism** |
| --- | --- | --- | --- | --- | --- | --- | --- | --- | --- | --- | --- | --- | --- |
| **Risk factors: ↓** |  |  |  |  |  |  |  |  |  |  |  |  |  |
| **Age** | 0.002 | 0.03 | <0.001 | <0.001 | n.s. | n.s. | n.s. | n.s. | n.s. | n.s. | 0.01 | n.s. | n.s. |
| **Gender** | 0.001 | n.s. | 0.02 | n.s. (0.06) | <0.001 | 0.03 | 0.02 | <0.001 | n.s. | <0.001 | 0.001 | <0.001 | n.s. |
| **Preoperative anaemia** | <0.001 | <0.001 | <0.001 | <0.001 | <0.001 | - | - | n.s. | n.s. | <0.001 | <0.001 | n.s. | n.s. |
| **Craniotomy** | <0.001 | n.s. | <0.001 | <0.001 | <0.001 | <0.001 | - | 0.01 | 0.005 | <0.001 | <0.001 | n.s. | n.s. |
| **RBC transfusion** | - | <0.001 | <0.001 | - | <0.001 | <0.001 | - | <0.001 | <0.001 | <0.001 | <0.001 | <0.001 | <0.001 |
| **RBC units** | - | <0.001 | <0.001 | - | <0.001 | <0.001 | - | <0.001 | 0.003 | <0.001 | <0.001 | <0.001 | 0.003 |

Online Resource 4 shows the results of the univariate analysis for risk factors on postoperative outcomes for intracerebral haemorrhage (ICH) patients; n.s.= not significant (with p-values ≤ 0.10 still explicitly displayed); ICH: Intracerebral haemorrhage, LOS: Length of in-hospital stay

**Online Resource 5): Outcomes in dependence of additional vasospasm in aneurysmal subarachnoid haemorrhage patients**

|  | Vasospasm (n= 672) | No Vasospasm (n= 4,336) | Univariate Comparison |
| --- | --- | --- | --- |
| Age (y) | 55.8 (± 0.5), 55.0 (47.0; 65.0), min= 21, max=91 | 58.3 (± 0.2), 58.0 (49.0; 68.0), min= 18, max=96 | P<0.001 |
| Gender | Female: 67.4%, n= 453/672  Male: 32.6 %; n= 219/672  Female: 67.4 %; n= 453/672 | Female: 60.8 %; n= 2,635/4,336  Male: 39.2 %; n= 1,701/4,336 | P= 0.001 |
| Preoperative anaemia | 22.8 % (19.2 % - 26.7 %); n= 113/496 | 29.0 % (27.6 % - 30.5 %); n= 1,105/3,808 | P= 0.004 |
| Postoperative anaemia | 87.7 % (84.9 % - 90.1 %); n= 584/666 | 79.8 % (78.6 % - 81.0 %); n= 3,314/4,152 | P<0.001 |
| Hospital-acquired anaemia | 87.2 % (83.4 % - 90.4 %); n= 334/383 | 75.6 % (73.9 % - 77.2 %); n= 2,000/2,645 | P<0.001 |
| Clipping | 31.7 % (28.2 % - 35.4 %); n= 213/672 | 36.8 % (35.4 % - 38.3 %); n= 1,596/4,336 | P= 0.01 |
| Coiling | 67.3 % (63.6 % - 70.8 %); n= 452/672 | 21.6 % (20.4 % - 22.9 %); n= 938/4,336 | P<0.001 |
| Craniotomy | 43.3 % (39.5 % - 47.1 %); n= 291/672 | 47.1 % (45.6 % - 48.6 %); n= 2,044/4,336 | n.s. (p= 0.06) |
| Additional ICH | 23.2 % (20.1 % - 26.6 %); n= 156/672 | 24.1 % (22.8 % - 25.4 %); n= 1,043/4,336 | n.s. |
| Length of stay (d) | 32.3 (± 0.9), 27.0 (19.0; 39.0) | 20.8 (± 0.3), 16.0 (8.0; 26.0) | P<0.001 |
| Mortality | 16.8 % (14.1 % - 19.9 %); n= 113/672 | 16.3 % (15.2 % - 17.4 %); n= 707/4,336 | n.s. |
| Renal failure | 5.2 % (3.7 % - 7.2 %); n= 35/672 | 6.0 % (5.3 % - 6.8 %); n= 261/4,336 | n.s. |
| Pulmonary embolism | 3.4 % (2.2 % - 5.1 %); n= 23/672 | 2.3 % (1.9 % - 2.8 %); n= 100/4,336 | n.s. |
| Pneumonia | 31.5 % (28.0 % - 35.2 %); n= 212/672 | 17.3 % (16.2 % - 18.5 %); n= 752/4,336 | P<0.001 |
| Sepsis | 13.1 % (10.6 % - 15.9 %); n= 88/672 | 7.9 % (7.1 % - 8.7 %); n= 341/4,336 | P<0.001 |
| Myocardial infarction | 2.2 % (1.3 % - 3.7 %); n= 15/672 | 1.7 % (1.4 % - 2.2 %); n= 75/4,336 | n.s. |
| Ischaemic stroke | 34.2 % (30.6 % - 38.0 %); n= 230 | 20.5 % (19.3 % - 21.8 %); n= 890/4,336 | P<0.001 |
| RBC transfusion | 40.9 % (37.2 % - 44.7 %); n= 275/672 | 28.1 % (26.8 % - 29.5 %); n= 1,220/4,336 | P<0.001 |
| RBC (units) / 1,000 patients | 2,573 (± 772), 0 (0; 2,000), min= 0, max=511,000 | 1,819 (± 99), 0 (0; 1,000), min= 0, max=118,000 | P<0.001 |

Online Resource 5 shows the outcomes in dependence of additional vasospasm in aneurysmal subarachnoid haemorrhage patients All values are represented either as mean (± SE), median (IQR) or as rate (95% CI) with total number, ICH: intracerebral haemorrhage
